# Bile and Serum Metabolomics in Living Donor Liver Transplantation: Exploratory Insights into Acute Rejection Biomarkers

**DOI:** 10.3390/metabo16040273

**Published:** 2026-04-17

**Authors:** Yuta Hirata, Yasunaru Sakuma, Hideo Ogiso, Taiichi Wakiya, Takahiko Omameuda, Toshio Horiuchi, Noriki Okada, Yukihiro Sanada, Yasuharu Onishi, Hironori Yamaguchi, Ryozo Nagai, Kenichi Aizawa

**Affiliations:** 1 Department of Surgery, Division of Gastroenterological, General and Transplant Surgery, Jichi Medical University, Tochigi 329-0498, Shimotsuke, Japan; 2 Department of Translational Research, Clinical Research Center, Jichi Medical University, Tochigi 329-0498, Shimotsuke, Japan; 3 Jichi Medical University, Tochigi 329-0498, Shimotsuke, Japan; 4 Clinical Pharmacology Center, Jichi Medical University Hospital, Tochigi 329-0498, Shimotsuke, Japan

**Keywords:** liver transplantation, acute rejection, biomarker, metabolomics

## Abstract

Background: Acute rejection remains a major complication following liver transplantation, yet reliable noninvasive biomarkers for its early prediction and diagnosis remain unidentified. This exploratory study characterized bile and serum metabolites associated with acute rejection in living donor liver transplantation using comprehensive metabolomic profiling combined with machine learning. Methods: Non-targeted metabolomics were performed on bile samples collected on post-operative day (POD) 1 (*n* = 38) and serum on POD 14 (*n* = 45) from liver transplant recipients. Partial least squares discriminant analysis-based variable selection was followed by logistic regression and least absolute shrinkage and selection operator models, which were evaluated via cross-validation in the discovery cohort to explore potential biomarkers for acute rejection. Results: A three-variable, bile-based model for predicting acute rejection achieved a mean cross-validated AUC of 0.872 (95% confidence interval: 0.814–0.930). Glycohyocholic acid and sulfolithocholylglycine were the main contributors. A nine-variable serum model for the Rejection Activity Index, including the change in γ-glutamyl transferase, showed a mean cross-validated R^2^ of 0.728 (95% confidence interval: 0.609–0.846), with methionine, creatine, and oxidized fatty acids contributing prominently. Conclusions: These findings suggest that metabolomic profiling combined with machine learning may provide candidate biomarkers for acute rejection after liver transplantation. However, given the exploratory nature of the study and the lack of external validation, the clinical utility of these metabolite signatures remains to be determined. Therefore, external validation in larger, independent cohorts will be required.

## 1. Introduction

Liver transplantation (LT) is the most effective curative treatment for end-stage liver disease with irreversible hepatic dysfunction. However, severe post-operative complications remain a significant challenge, limiting the long-term success of LT. Short-term complications include graft dysfunction, acute rejection (AR), infections, and systemic disorders. Despite advanced immunosuppressive therapies, AR remains one of the most common and challenging complications in transplant recipients, occurring with a frequency of approximately 10% between 5 and 30 days post-transplant [1]. Notably, the proportion of living donor liver transplants among total liver transplants is approximately 25% worldwide, whereas it exceeds 80% in Japan [2].

The main symptoms of AR include fever, elevated serum bilirubin, aspartate aminotransferase, alanine aminotransferase, gamma-glutamyl transferase, eosinophil count, thrombocytopenia, and ascites. However, these clinical and laboratory findings alone are insufficient to confirm AR. A definitive diagnosis requires histopathological examination via liver biopsy. Following liver transplantation, liver biopsy is considered the gold standard for evaluating organ rejection. However, this procedure is invasive and carries risks of clinical complications. Additionally, accuracy of pathological diagnosis is influenced by factors such as pathologist experience and quality of the biopsy sample. For early prediction of AR, which typically manifests after POD5, bile collected on POD1 offers a unique advantage. Bile is in direct contact with the grafted liver from the immediate postoperative period and may reflect early graft injury, including ischemia–reperfusion injury and initial alloimmune responses, before clinical symptoms or laboratory abnormalities appear. In addition, acute rejection is known to affect primarily the biliary epithelium, and early immune-mediated injury to bile ducts may occur before clinically apparent rejection, potentially influencing bile acid composition at an early postoperative stage.

For prediction and diagnosis of AR, non-invasive methods and use of biomarkers are widely recognized as advantageous strategies [3,4]. For example, biomarkers such as donor-specific, cell-free DNA derived from transplanted cells, microRNAs, and other molecular indicators have been investigated for monitoring graft injury [5,6,7,8]. Similarly, metabolomic approaches have been explored as potential tools for identifying rejection-associated markers [9,10]. Bile acid profiling, in particular, has mainly been used to assess overall graft function after liver transplantation [11,12], rather than to predict future rejection events. Although metabolomics have been used to evaluate graft dysfunction and rejection, use of metabolomic data specifically to predict rejection, remains at an early, exploratory stage. In this study, we performed non-targeted metabolomic analysis of stored post-transplant bile and serum samples obtained from liver transplant recipients to detect candidate metabolite markers associated with AR and its histological severity. Recent advances in multivariate analysis and machine learning have enabled extraction of complex metabolic patterns that cannot be captured by conventional univariate statistical methods [13,14]. However, such approaches are also susceptible to overfitting, particularly in high-dimensional datasets with limited sample sizes. To partially address this risk in the present exploratory study, internal cross-validation, permutation testing, and bootstrapping were applied to the final models; however, due to practical constraints, these validations were not performed in a nested fashion. Consequently, machine learning-based modeling was used primarily to nominate promising metabolite candidates and to evaluate their preliminary predictive value in the discovery cohort, while acknowledging the remaining possibility of overfitting. External validation in larger, independent cohorts will be required before any clinical utility can be established.

## 2. Materials and Methods

### 2.1. Ethical Approval

This study was conducted in accordance with the Declaration of Helsinki and approved by the Institutional Ethics Committee of Jichi Medical University (Approval Code: 20-008, Approval Date: 27 November 2020). Informed consent was obtained from all patients or their legal guardians.

### 2.2. Chemicals and Reagents

Details are provided in the Supplemental Materials.

### 2.3. Specimens and Grouping

In a living donor LT, an external biliary fistula tube is inserted, and bile is collected through this tube. Bile and serum samples were collected after LT and stored at −80 °C until analysis. Herein, bile collected on post-operative day (POD)1 is termed POD1-bile, and serum collected on POD14 is denominated POD14-serum. Liver biopsy was performed to diagnose acute rejection in cases presenting liver dysfunction or unexplained ascites. The median time to onset of acute rejection was 10 days after LT (range, 5–33 days). Types of AR included T cell-mediated rejection in 13 cases and antibody-mediated rejection in 1 case. In this study, AR severity was assessed using the Rejection Activity Index (RAI) according to standard histopathological criteria. Biopsy was performed only when rejection was clinically suspected, and observed RAI values in the rejection group ranged from 3.5 to 9. For the non-rejection group, in which biopsy was not performed, an RAI value of 2 was assigned for regression analysis, assuming minimal histological rejection activity. This assignment is a strong assumption, and sensitivity analyses with alternative values were not conducted in this exploratory study. These results should therefore be interpreted with caution, as the aim of this analysis was to explore potential candidate markers rather than to establish a predictive model. Baseline recipient characteristics are summarized in Table 1 and Table 2. Since marked changes in bile metabolites may have occurred mainly between POD7 and POD14, POD1-bile was used to analyze the predictability of AR onset, while POD14-serum was used for correlation analysis with RAI.

### 2.4. Immunosuppressive Therapy

Tacrolimus (Tac) and methylprednisolone (MP) were used as the standard postoperative immunosuppressive therapy. The target trough level of Tac was 15–20 ng/mL during the first week, 8–12 ng/mL during the first month, 5–8 ng/mL during the first six months, 3–5 ng/mL during the first year, and 2–4 ng/mL thereafter. MP was administered at an initial dose of 20 mg/kg intravenously on the morning of the operation and before graft reperfusion. Thereafter, the MP dose was gradually decreased to 3 mg/kg/day on POD 1, 0.5 mg/kg/day on POD 7, 0.25 mg/kg/day at 1-month post-surgery, and discontinued within one year, except in patients for whom immunosuppression could not be maintained at the lower dose. Mycophenolate mofetil was used when more potent immunosuppression was required, for instance, in ABO-incompatible recipients, in patients with steroid-resistant rejection episodes, and in patients with liver dysfunction after cessation of MP therapy, in patients with dose reduction in Tac and MP due to an adverse event.

### 2.5. Diagnosis and Treatment of AR

In our department, we perform liver biopsy to diagnose AR in cases presenting either liver dysfunction or unknown ascites. Yamada et al. (2012) showed that sinusoidal obstruction syndrome due to AR led to increased ascites [15]. AR was assessed according to Banff criteria [16]. RAI was defined so that three features, portal inflammation, bile duct inflammation/damage, and venular inflammation, could be more critically evaluated and scored semi-quantitatively on a 0 to 3 (mild, moderate, and severe) scale [17]. We administered steroid pulse therapy to the AR group.

### 2.6. Sample Preparation

Sample preparation and liquid chromatography quadrupole time-of-flight mass spectrometry (LC-QTOF-MS) analysis were performed by modifying a previous method [18,19]. Bile metabolites were extracted as follows. 50 µL of bile were mixed with 200 µL of methanol (MeOH) at room temperature, vigorously shaken, and allowed to stand on ice for 30 min. After shaking again, the mixture was centrifuged (14,000× *g*, 10 min, 4 °C) to obtain supernatant. To 10 µL of supernatant, 490 µL of internal standard solution were added, mixed, and centrifuged in the same manner to obtain 200 µL of supernatant. The internal standard solution contained 5 mM L-phenylalanine-13C6, 0.25 mM sulfadimethoxine, 0.5 mM fusidic acid, 0.5 mM PE(34:1)d31 in ultra-pure water/MeOH/2-propanol (4:4:2, *v*/*v*/*v*).

Serum metabolites were extracted as follows. 20 µL of serum were mixed with 80 µL of MeOH at room temperature, vigorously shaken, and allowed to stand on ice for 30 min. After a second shaking, the mixture was centrifuged (14,000× *g*, 10 min, 4 °C) to obtain supernatant. To 40 µL of this supernatant, 160 µL of the internal standard solution were added and centrifuged in the same manner to obtain 180 µL of supernatant, which were analyzed using liquid chromatography quadrupole time-of-flight mass spectrometry (LC-QTOF-MS).

### 2.7. LC-QTOF-MS Analysis

Non-targeted metabolomics were conducted using an LC-QTOF-MS system (Shimadzu LCMS9030; Shimadzu Corporation, Kyoto, Japan). An Accura Triart C_18_ (2.1 × 100 mm, 1.9 µm; YMC, Kyoto, Japan) was used for metabolite separation. Mobile phase A consisted of 0.1% (*v*/*v*) formic acid in ultra-pure water and mobile phase B consisted of 0.1% (*v*/*v*) formic acid in MeOH/acetonitrile (1:1, *v*/*v*). Metabolites were eluted from the column with the following gradient program at a flow rate of 0.32 mL/min. Starting at 0% B, initial conditions were maintained for 1 min, increased to 5% B between 1 and 3 min, increased to 90% B between 3 and 9 min, increased to 100% B between 9 and 11 min, maintained at 100% B from 11 to 15 min, returned to 0% B between 15 and 15.5 min, and maintained at 0% B from 15.5 to 20 min. The time required to measure one sample was 20 min. The column temperature was 45 °C and the sample cooler temperature was 4 °C.

One and 2 µL of sample were injected in positive (pos) and negative (neg) ion modes, respectively. Mass spectrometry (MS) data were acquired separately in each ion mode from 70 to 900 *m*/*z* using electrospray ionization. MS parameters used default settings as follows: interface voltage of 4.5 kV (pos) and −3.5 kV (neg), interface temperature of 300 °C, nebulization gas flow of 3 L/min, heating gas flow of 10 L/min, drying gas flow of 3 L/min, heat block temperature of 400 °C, and DL temperature of 250 °C. Before sample measurements, *m*/*z* values were calibrated using a calibrant (ESI Tuning Mix for Ion Trap, Supelco, Bellefonte, PA, USA), so that mass accuracy remained within 10 ppm throughout sample measurements. A mixture of representative samples among those tested was used as a quality control (QC) reference. The QC sample was injected six times in total at the beginning, middle, and end of the batch to check stability of detected compounds during measurements. Analytical stability was confirmed by ensuring that the relative standard deviation (RSD) of peak intensities for representative compounds in the QC samples remained below 20%. Peak intensities were directly used as relative abundance based on a fixed injection volume per sample, without additional normalization such as internal standards or total signal scaling. Relative abundance of each peak was evaluated by intensity of the parent ion (MS1) in a full-scan mode. Additional measurements were also made in data-dependent auto MS/MS mode to obtain fragmentation ions (MS2) necessary for structure estimation. Fragmentation was performed using argon as a collision gas at a collision energy of 30 eV with a spread of 15 eV. MS2 spectra were measured simultaneously for the top five ions with an *m*/*z* range between 50 and 900, surpassing an intensity threshold of 2000 (pos) and 500 (neg). Data were collected using LabSolutions software version 5.118 for the LCMS9030 (Shimadzu). Peak intensities below the detection threshold were recorded as zero and were not treated as missing data.

After converting acquired data into xml files, peak detection and alignment were performed using MS-DIAL (ver. 5.1) to assign peak IDs. Compound names were then annotated using both MS-DIAL and MS-FINDER (ver. 3.56) [20,21]. As necessary, MS1 ions were searched against the HMDB metabolite database (ver. 5.0, https://www.hmdb.ca/ accessed on 17 April 2025), MassBank of North America (https://mona.fiehnlab.ucdavis.edu/ accessed 25 August 2025), MetFrag (https://msbi.ipb-halle.de/MetFragBeta/ accessed on 3 September 2025), and the LIPID MAPS lipid database (https://lipidmaps.org/databases accessed on 25 August 2025). Annotation was carried out according to a method previously reported [18]. Metabolites were annotated at two confidence levels. Compounds were assigned as Rank A when the MS^2^ spectrum showed reasonable agreement with a database entry and the retention time was consistent with the expected value. If a reliable MS^2^ spectrum could not be obtained, but the MS^1^ mass matched the sole metabolite registered in HMDB, the compound was also annotated as Rank A. Rank B annotation was assigned when MS^1^ and/or MS^2^ spectra matched database records, but could not be resolved to a single compound due to multiple candidate hits, or when the metabolite was uncommon and the observed retention time was inconsistent with the predicted value. In such cases, the most likely candidate was annotated as Rank B and denoted with a delta symbol (Δ) to indicate its provisional assignment.

### 2.8. Metabolite Selection and Model Construction

**Figure 1:**
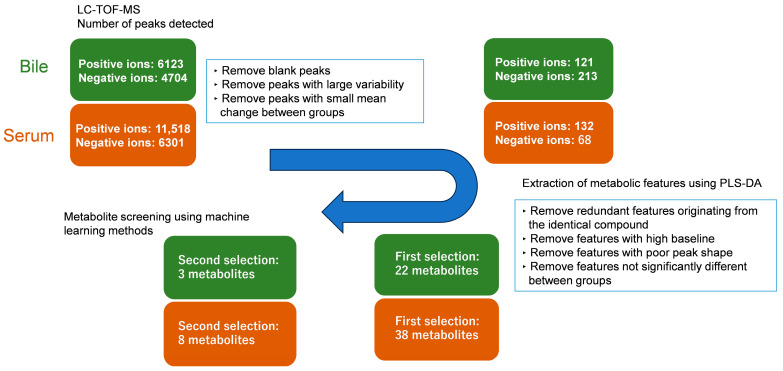
Screening Procedure for Bile and Serum Metabolites in Non-Targeted Metabolomics. Metabolic features in bile (green) and serum (brown) samples were selected using partial least squares-discriminant analysis (PLS-DA) and machine learning methods, including LASSO regression and logistic regression, to identify biomarkers for acute rejection. Bile (rejection/non-rejection): *n* = 13/25; serum (rejection/non-rejection): *n* = 14/31.

Selection and refinement of metabolites were performed in a two-step, exploratory procedure (Figure 1). Over 10,000 peaks were detected by LC-QTOF-MS in both bile and serum metabolomic analyses. Prior to multivariate analysis, features with poor reproducibility or negligible alteration were excluded to improve analytical reliability. Specifically, peaks were removed if they met either of the following criteria in the non-rejection group samples: (i) RSD > 200%, or (ii) average intensity change rate < 30% relative to the non-rejection group mean.

The remaining features were subjected to partial least squares discriminant analysis (PLS-DA) using MetaboAnalyst 6.0 (https://www.metaboanalyst.ca/MetaboAnalyst/ accessed on 23 August 2025). To improve the reliability of candidate selection, four independent PLS-DA models were constructed using different ionization modes and scaling methods (positive/negative ion mode × autoscaling/Pareto scaling). From each model, the top 30–40 features with the highest Variable Importance in Projection (VIP) scores were selected. After merging the candidate lists, removing duplicates, and excluding obvious contaminants by visual inspection, 22 metabolic features from bile and 38 from serum were retained as primary candidates.

At this stage, compound annotation was not strictly performed, and all features were defined solely by their unique peak IDs. It should be noted that, given the limited number of rejection events (13 in bile and 14 in serum), this VIP-based selection step was conducted on the full dataset as an exploratory dimensionality-reduction procedure and not as definitive biomarker identification. Because feature selection was performed prior to subsequent model validation, the pipeline does not constitute a fully nested modeling framework. Consequently, the reported predictive performance may carry optimistic bias, and overfitting risk cannot be completely excluded despite internal validation on the final models.

For these selected features, normalized data matrices were generated from the raw peak intensity values by scaling relative changes in peak intensities using the same procedure as in the initial preprocessing. These normalized data served as input for subsequent machine-learning modeling.

In the second stage, machine learning-based modeling was performed. For bile metabolites, a binary prediction model discriminating between rejection and non-rejection was constructed using least absolute shrinkage and selection operator (LASSO) regression. For serum metabolites, continuous RAI prediction models were developed using LASSO regression. Ultimately, 3 to 8 metabolites were selected for each final model. All modeling was conducted using Gofard (ver. 1.2) and R (ver. 4.4.2). Given the small event numbers and methodological constraints, all models were regarded as exploratory.

### 2.9. Model Validation

Due to the limited sample size, no independent validation cohort was available. Model parameters were optimized on the full dataset for feature selection, while final performance was evaluated exclusively using cross-validation in the discovery cohort. Internal validation was therefore performed using 4-fold cross-validation within the discovery cohort 15. Importantly, initial metabolite feature selection by PLS-DA was conducted on the full dataset, and cross-validation was applied only to the final selected models. This non-nested approach carries a risk of optimistic bias due to potential information leakage from the feature selection step. For the binary AR prediction model, 4-fold cross-validation was repeated four times (16 test sets in total). For the continuous RAI prediction model, 4-fold cross-validation was performed once. Model stability was further evaluated by leave-one-out cross-validation (LOOCV), permutation testing (*n* = 1000), and 0.632+ bootstrap resampling (*n* = 1000). All validation procedures were conducted in R (ver. 4.4.2). It should be noted that these internal validation procedures provide only an estimate of model consistency within the discovery cohort and do not represent external generalizability. Therefore, the reported performance should be interpreted as exploratory, and independent external validation will be required to establish clinical utility.

### 2.10. Statistical Analysis

Continuous variables were summarized by median and interquartile range, and categorical variables by counts and percentages. The Mann–Whitney U test was used for comparisons of continuous variables between groups, and Fisher’s exact test was applied to categorical variables. A two-sided *p*-value < 0.05 was considered statistically significant. Statistical analyses were performed using SPSS version 30.0 (IBM Corp., Armonk, NY, USA).

## 3. Results

### 3.1. Baseline and Perioperative Characteristics of POD1-Bile and POD14-Serum Cohorts

Baseline characteristics of patients with POD1-bile samples are summarized in Table 1. No significant differences were observed between the rejection and non-rejection groups in demographic, clinical, or perioperative variables, including cold and warm ischemia times. Characteristics of patients with POD14-serum samples are presented in Table 2. In the POD1-bile cohort, no significant differences were found in baseline or perioperative variables. However, the rejection group showed significantly higher serum TBIL, DBIL, and ALT levels at POD14 compared with the non-rejection group (*p* = 0.029, *p* = 0.016, and *p* = 0.012, respectively).

### 3.2. Development of a Bile Metabolomic Model for Rejection Prediction

**Figure 2:**
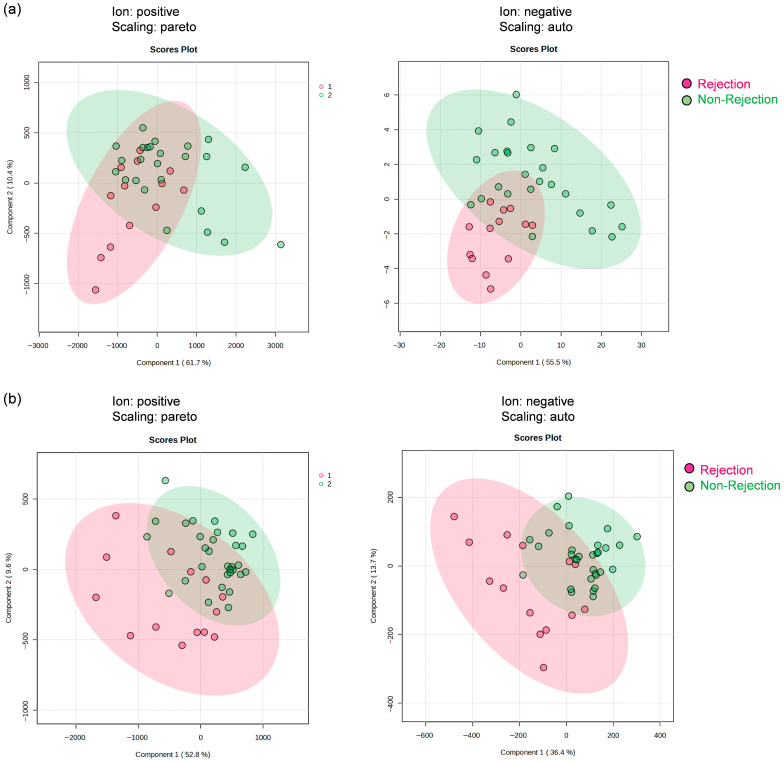
PLS-DA score plots of bile and serum metabolomic data for feature selection. Score plots from partial least squares discriminant analysis (PLS-DA) of positive- and negative-ion mode datasets. PLS-DA was performed separately on POD1-bile and POD14-serum samples to select metabolic features discriminating the rejection group (Group 1) and the non-rejection group (Group 2). Panel (**a**) shows the score plots for POD1-bile data, and panel (**b**) shows those for POD14-serum data. PLS-DA was conducted using MetaboAnalyst (ver. 6.0; https://www.metaboanalyst.ca/).

In a retrospective discovery cohort study, non-targeted metabolomic analysis was performed on preserved bile samples collected on POD1 from 38 liver transplant patients. Using their metabolomic data, a group comparison was conducted between the rejection and non-rejection groups, comprising 13 and 25 samples, respectively. A PLS-DA-based exploratory screening approach was used to select candidate metabolic features distinguishing these groups. Significant metabolic features were evaluated based on peak IDs and their intensities in positive and negative ion modes. Representative PLS-DA score plots for rejection versus non-rejection groups are shown in Figure 2a. Because important metabolic features with high VIP scores included multiple signals derived from the same compound and features derived from blanks, these were excluded, and only original metabolic features were retained. As a result of this initial PLS-DA screening, 22 metabolic features were retained from bile samples as primary candidates.

**Figure 3:**
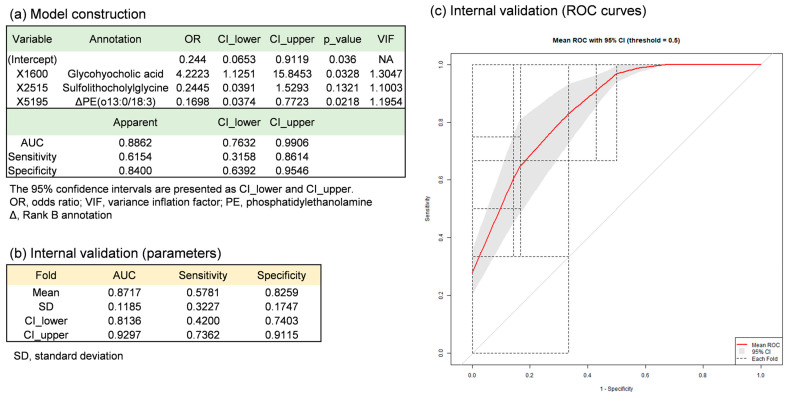
Binary predictive model for rejection using POD1-bile metabolomic data in the discovery cohort. Twenty-two metabolites were first selected by PLS-DA in the discovery cohort. Using these 22 metabolites, variable selection was performed with LASSO logistic regression on the full discovery cohort, resulting in a final optimized model with three metabolites. Panel (**a**) shows parameters of the optimized three-metabolite model fitted to all samples (model construction). Panel (**b**) shows parameters of the same model with the three fixed metabolites evaluated by 4-fold cross-validation (internal validation). Panel (**c**) shows the ROC curves from the 4-fold cross-validation. Sixteen individual ROC curves (black dotted lines) from four iterations are overlaid, together with the mean ROC curve (solid red line). Mean values and 95% confidence intervals (95% CI) of AUC, sensitivity, and specificity derived from the cross-validation are also presented. Because feature selection was conducted outside the cross-validation loop (non-nested approach) due to the limited sample size, the cross-validated performance may be subject to optimistic bias from information leakage. Model building and validation were performed using R (ver. 4.4.2).

To further refine the bile metabolite panel, LASSO logistic regression was applied to the 22 primary candidate features measured in the full bile cohort (*n* = 38). This second-stage analysis generated several candidate prediction models. The three-metabolite model was selected because it offered comparable performance with the smallest number of variables (Figure 3a, Table 3, and Supplementary Table S1).

Given the limited sample size, internal consistency was assessed using 4-fold cross-validation repeated four times (16-fold in total) for the final three-metabolite model. The mean AUC across replicates was 0.872 (95% confidence interval: 0.814–0.930), with a mean sensitivity of 0.578 (95% confidence interval: 0.420–0.736) and a mean specificity of 0.826 (95% confidence interval: 0.740–0.912) (Figure 3b,c). In addition, leave-one-out cross-validation (LOOCV), permutation testing (*n* = 1000), and 0.632+ bootstrap resampling (*n* = 1000) were performed. The model yielded an AUC of 0.831 by LOOCV (Supplementary Figure S1). The permutation test showed an observed AUC of 0.886 with an empirical *p*-value of 0.002, and the 0.632+ bootstrap analysis gave a 95% confidence interval of 0.806–0.898 for the AUC (Supplementary Figure S1).

Compound annotation was performed for metabolites included in the three-metabolite model using MS-DIAL, MS-FINDER, and MetFrag. Two metabolites were annotated as glycohyocholic acid and sulfolithocholylglycine at Rank A based on agreement of MS^2^ spectra with database entries and consistent retention times (Supplementary Figure S2). One additional metabolite was classified as Rank B (provisional assignment) due to partial MS^2^ agreement with database records together with uncertainty in retention time and metabolite prevalence. All annotations are putative and should not be regarded as definitive identifications.

These results should be interpreted with caution, as the observed performance declined compared with model selection-based estimates, and confidence intervals remain relatively wide, reflecting the limited sample size and exploratory nature of the analysis.

### 3.3. Development of a Serum Metabolomic Model for RAI Prediction

In a retrospective discovery cohort study, non-targeted metabolomic analysis was performed on preserved serum samples collected on POD14 from 45 liver transplant patients. Using their metabolomic data, a group comparison was conducted between rejection and non-rejection groups, comprising 14 and 31 samples, respectively. A PLS-DA-based exploratory screening approach was used to select candidate metabolic features distinguishing these groups. Significant metabolic features were evaluated based on peak IDs and their intensities in positive and negative ion modes. Representative PLS-DA score plots are shown in Figure 2b. After excluding duplicated, blank-derived, or non-original features, 38 metabolic features were retained as primary candidates from the serum samples.

**Figure 4:**
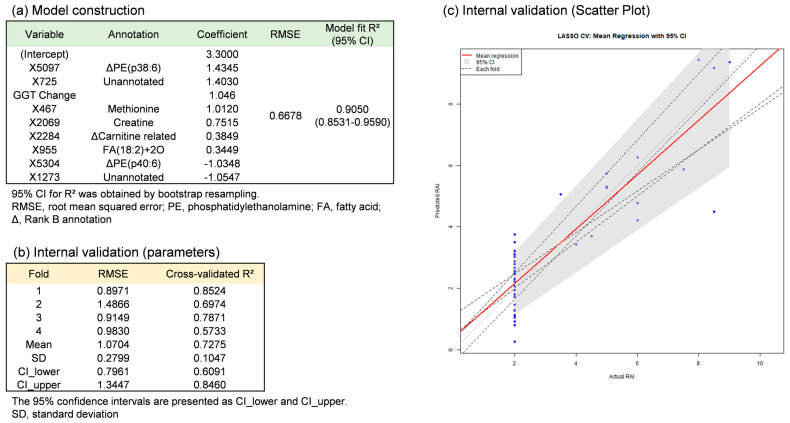
RAI predictive model for rejection using POD14-serum metabolomic data in the discovery cohort. Thirty-eight metabolites were first selected by PLS-DA in the discovery cohort. Using these 38 metabolites together with biochemical parameters, variable selection was performed with LASSO regression on the full discovery cohort, resulting in a final optimized model consisting of eight metabolites and one biochemical parameter (nine variables in total). Panel (**a**) shows parameters of the optimized nine-variable model fitted to all samples (model construction). Panel (**b**) shows parameters of the same model with the nine fixed variables evaluated by 4-fold cross-validation (internal validation). Panel (**c**) shows regression lines from the 4-fold cross-validation. Four individual regression lines (black dotted lines) are overlaid, together with the mean regression line (solid red line). The mean and 95% confidence intervals (95% CI) of predicted RAI values are also presented. Because feature selection was conducted outside the cross-validation loop (non-nested approach) due to the limited sample size, the cross-validated performance may be subject to optimistic bias from information leakage. Model building and validation were performed using R (ver. 4.4.2).

LASSO regression analysis was applied to develop continuous-variable prediction models for RAI using these 38 metabolites incorporating hematological and biochemical parameters. To address potential confounding by immunosuppressive therapy, methylprednisolone dosage was additionally included as a covariate in the LASSO analysis. The final model was constructed using combinations of 9 variables (Figure 4a, Table 4, and Supplementary Table S2). Notably, the methylprednisolone dosage was not selected in the final model. The 9-variable model included the change in γ-glutamyl transferase (GGT) between POD14 and POD1.

Similarly, although the model demonstrated reasonable internal performance, the decline in performance upon cross-validation and the associated uncertainty indicate that these findings should be interpreted cautiously.

Subsequently, internal validation was performed using 4-fold cross-validation in the discovery cohort. For the four test datasets obtained by 4-fold cross-validation, the nine-variable LASSO model achieved a mean cross-validated R^2^ of 0.728 (95% confidence interval: 0.609–0.846) (Figure 4b,c). In addition, leave-one-out cross-validation (LOOCV), permutation testing (*n* = 200), and 0.632+ bootstrap resampling (*n* = 1000) were performed. The model yielded an R^2^ of 0.814 by LOOCV. The permutation test showed an observed R^2^ of 0.752 with an empirical *p*-value of 0.000, and the 0.632+ bootstrap analysis gave a 95% confidence interval of 0.739–0.896 for the R^2^ (Supplementary Figure S3).

Consistent with the bile metabolomic approach, compound annotation was performed for the 8 metabolites included in the LASSO model. Five metabolites were annotated as methionine, oxidized fatty acid (FA(18:2)+2O), and creatine with Rank A based on agreement of MS^2^ spectra with database entries and consistent retention times (Table 4 and Supplementary Figure S4). Three additional metabolites were classified as Rank B (provisional assignments) due to uncertainty in retention time, low metabolite prevalence, or lack of MS^2^ spectra. The remaining two metabolites could not be annotated.

### 3.4. Individual Evaluation of Selected Metabolites

The predictive models described above were constructed using combinations of metabolites selected through multivariate analysis. Ultimately, the three metabolites in the bile model and the eight in the serum model were evaluated individually, but some did not show statistically significant differences between groups. This suggests that their relevance lies in their combination rather than their isolated effects. ROC curves and box plots for the three bile metabolites in the binary prediction model for rejection are shown in Supplementary Figure S5. Glycohyocholic acid (Rank A annotation), sulfolithocholylglycine (Rank A annotation), and ether-linked phosphatidylethanolamine (o13:0/18:3) (Rank B annotation) had AUCs of 0.611, 0.588, and 0.769, respectively. Scatter plots with regression lines and violin plots for the eight serum metabolites in the continuous variable prediction model for RAI are shown in Supplementary Figure S6. Oxidized fatty acid (FA(18:2)+2O, Rank A annotation), carnitine-related compound (Rank B annotation), and methionine (Rank A annotation) were relatively highly correlated, with Spearman’s correlation coefficients (ρ) of 0.4616 and 0.4428, respectively (Supplementary Figure S6).

## 4. Discussion

### 4.1. Study Overview and Limitations

In this retrospective discovery cohort study, non-targeted metabolomic analyses were performed using bile samples collected on POD1 and serum samples collected on POD14. The aim was to explore metabolites in POD1 bile that might predict subsequent AR, which occurs in approximately 10% of patients between 5 and 30 days after liver transplantation, and metabolites in POD14 serum that might reflect the severity of rejection as assessed by the RAI. Primary limitations of this study include the small number of rejection events (13 in bile and 14 in serum), the lack of external validation, potential confounding effects of steroid pulse therapy in the rejection group, and inclusion of several unannotated or provisionally annotated (Rank B with Δ) metabolites in the final models. Predictive performance was assessed solely by internal cross-validation. Moreover, metabolite feature selection was performed using PLS-DA on the full dataset prior to model construction, and cross-validation was applied only to the final selected models. As a result, performance metrics are likely optimistically biased. These findings should therefore be regarded strictly as exploratory and hypothesis-generating. The primary aim of this study was to nominate candidate metabolites worthy of further investigation in larger, independent cohorts. In addition, although T cell-mediated rejection and antibody-mediated rejection have distinct pathophysiological mechanisms, antibody-mediated rejection is characterized by donor-specific antibodies and associated bile duct injury [22]. Due to the limited number of antibody-mediated rejection cases (*n* = 1), separate analysis was not feasible; therefore, these were analyzed as a single group, and the findings should be interpreted as reflecting general rejection-associated metabolic alterations rather than subtype-specific differences.

### 4.2. Bile Metabolites for Early Prediction of Acute Rejection

In the binary prediction model for AR, two glycine-conjugated bile acids, glycohyocholic acid and sulfolithocholylglycine, both annotated at Rank A, were selected as key variables (Figure 3). In POD1 bile from rejection cases, glycohyocholic acid levels increased approximately two-fold, whereas sulfolithocholylglycine levels decreased 0.4-fold compared with non-rejection cases (Table 3). No significant differences in baseline clinical characteristics were observed between the groups (Table 1). Because AR typically occurs between PODs 5 and 14, these findings suggest the possibility that bile metabolites measured on POD1 may help predict rejection risk before clinical onset. In addition, AR affects primarily the biliary epithelium, and early immune-mediated injury to bile ducts may occur before clinically apparent rejection, potentially influencing bile acid composition at an early postoperative stage. Bile acids such as glycohyocholic acid and sulfolithocholylglycine have been implicated in immune regulation via FXR- and TGR5-mediated signaling pathways [23,24], although direct mechanistic links in the context of graft rejection remain to be identified.

### 4.3. Serum Metabolites Reflecting Rejection Activity

The continuous-variable prediction model for the Rejection Activity Index (RAI) with the highest internal performance included the change in GGT with serum methionine and creatine (both Rank A) and seven other metabolites (Figure 4). Serum methionine and creatine levels were 1.6-fold and 2.4-fold higher, respectively, in the rejection group, with Spearman correlations to RAI of 0.437 and 0.384 (Table 4 and Supplementary Figure S6). These correlations were stronger than that of the GGT change alone (ρ = 0.226). To address the potential confounding effect of immunosuppressive therapy, methylprednisolone dosage was included as a covariate in the LASSO analysis. However, it was not selected in the final model, suggesting that identified metabolites may be less influenced by steroid dosage and more likely reflect changes associated with graft rejection. However, a potential confounding effect of corticosteroid administration cannot be completely excluded, and these results should be interpreted with caution. Elevated serum methionine may reflect impaired methionine metabolism associated with reduced liver function [25], while increased GGT is commonly linked to biliary injury [26,27].

A random forest regression model was also evaluated for RAI prediction. However, its performance declined markedly under cross-validation, whereas the LASSO model maintained more stable performance. Consequently, the LASSO model was selected as the final model. These alterations may be associated with graft rejection; however, whether the metabolite panel can improve upon or replace the current gold standard RAI requires further investigation in larger cohorts.

## 5. Conclusions

This study identifies bile and serum metabolite signatures that are associated with the onset and histological severity of acute rejection following liver transplantation, highlighting the potential of metabolomics to capture early graft-related biological changes that are not detectable by conventional clinical parameters. These findings provide a basis for developing noninvasive strategies for risk stratification and disease monitoring in transplant recipients. However, given the exploratory design, potential optimistic bias in model performance, and the lack of external validation, these results should be interpreted with caution and require confirmation in independent cohorts.

## Figures and Tables

**Table 1:** Baseline characteristics of recipients with POD1 bile samples.

Variable	Rejection Group (*n* = 13)	Non-Rejection Group (*n* = 25)	*p* Value
Sex (Male/Female)	5/8	10/15	0.939
Age (years)	57 (31–60)	52 (1–68)	0.196
Original diseases (*n*)			-
Metabolic dysfunction-associated steatohepatitis	4	9	0.520
Biliary atresia	1	6	0.221
Alcohol liver cirrhosis	2	3	0.791
Primary biliary cholangitis	2	2	0.424
Primary sclerosing cholangitis	2	2	0.424
Others	2	3	0.791
Body weight (kg)	61.4 (43.6–74.0)	57.7 (7.8–88.0)	0.470
PELD or MELD	16.4 (5.2–41.5)	11.7 (0–39.2)	0.280
ABO compatibility (*n*)			-
Identical	6	17	0.169
Compatible	5	5	0.200
Incompatible	2	3	0.791
GV/SLV (%)	50.6 (35.1–70.6)	51.1 (31.2–85.6)	0.893
GRWR (%)	1.0 (0.7–1.3)	1.0 (0.6–2.8)	0.954
Operative time (min)	689 (584–1088)	712 (454–1229)	0.770
Blood loss (mL/kg)	76.2 (9.2–293.8)	89.8 (9.9–1112.4)	0.988
CIT (min)	78 (39–245)	70 (32–271)	0.275
WIT (min)	49 (33–71)	52 (35–73)	0.218
Donor age (years)	37 (23–61)	42 (20–59)	0.914
POD1 TBIL (mg/dL)	4.87 (2.5–13.5)	4.7 (1.9–10.79)	0.988
POD1 DBIL (mg/dL)	1.49 (0.18–5.08)	1.23 (0.22–3.99)	0.723
POD1 AST (U/L)	290 (124–815)	284 (89–1253)	0.939
POD1 ALT (U/L)	249 (83–1067)	219 (75–1506)	0.854
POD1 GGT (U/L)	38 (14–116)	34 (15–232)	0.580
POD1 Tacrolimus trough level	6.8 (3.4–11.0)	6.7 (1.3–18.0)	0.817
POD1 Mycophenolate mofetil dosage (mg/kg/day)	7.1 (0–7.4)	7.8 (0–10.6)	0.175
POD1 Methylprednisolone dosage (mg/kg/day)	2.9 (1.4–3.1)	2.8 (1.4–3.2)	0.700

Abbreviations: PELD, pediatric end-stage liver disease; MELD, model for end-stage liver disease; GV/SLV, graft volume to standard liver volume; GRWR, graft-to-recipient weight ratio; CIT, cold ischemia time; WIT, warm ischemia time; POD, postoperative day; ABO, blood type compatibility. Values are presented as median (interquartile range) for continuous variables and number for categorical variables. *p* values were calculated using the Mann–Whitney U test for continuous variables and Fisher’s exact test for categorical variables.

**Table 2:** Baseline characteristics of recipients with POD14 serum samples.

Variable	Rejection Group (*n* = 14)	Non-Rejection Group (*n* = 31)	*p* Value
Sex (Male/Female)	5/9	12/19	0.873
Age (years)	55 (31–66)	51 (0–68)	0.148
Original diseases (*n*)			-
Metabolic dysfunction-associated steatohepatitis	4	9	0.633
Biliary atresia	1	9	0.102
Alcohol liver cirrhosis	3	4	0.878
Primary biliary cholangitis	2	2	0.366
Primary sclerosing cholangitis	2	2	0.366
Others	2	5	0.626
Body Weight (kg)	60.1 (43.6–74.0)	56.8 (3.1–88.0)	0.462
PELD or MELD	15.7 (5.2–41.5)	11.7 (0–39.2)	0.391
ABO compatibility (*n*)			-
Identical	6	22	0.072
Compatible	5	5	0.142
Incompatible	3	4	0.878
GV/SLV (%)	50.7 (35.1–70.6)	50.2 (31.2–137.5)	0.270
GRWR (%)	1.0 (0.7–1.4)	1.0 (0.6–6.0)	0.221
Operative time (min)	695 (584–1088)	735 (454–1229)	0.455
Blood loss (mL/kg)	81.8 (9.2–293.8)	89.8 (9.9–1112.4)	0.923
CIT (min)	76 (39–245)	75 (32–271)	0.411
WIT (min)	47 (33–71)	52 (35–116)	0.120
Donor age (years)	37 (23–61)	42 (20–67)	0.912
POD14 TBIL (mg/dL)	4.85 (1.37–17.99)	1.89 (0.38–15.39)	0.029
POD14 DBIL (mg/dL)	3.01 (0.44–14.54)	0.74 (0.07–11.96)	0.016
POD14 AST (U/L)	36.5 (18–172)	30 (11–108)	0.138
POD14 ALT (U/L)	113 (16–834)	49 (14–395)	0.012
POD14 GGT (U/L)	183 (36–962)	129 (41–326)	0.281
POD14 Tacrolimus trough level	12.8 (3.3–18.7)	12.0 (4.6–20.5)	0.548
POD14 Mycophenolate mofetil dosage (mg/kg/day)	20.6 (0–31.1)	16.2 (0–27.5)	0.625
POD14 Methylprednisolone dosage (mg/kg/day)	2.1 (0.3–10.6)	0.2 (0–0.5)	< 0.001

Abbreviations: PELD, pediatric end-stage liver disease; MELD, model for end-stage liver disease; GV/SLV, graft volume to standard liver volume; GRWR, graft-to-recipient weight ratio; CIT, cold ischemia time; WIT, warm ischemia time; POD, postoperative day; ABO, blood type compatibility. Values are presented as median (interquartile range) for continuous variables and number for categorical variables. *p* values were calculated using the Mann–Whitney U test for continuous variables and Fisher’s exact test for categorical variables.

**Table 3:** Characteristics of the three bile metabolites selected in the final logistic regression model for predicting rejection.

ID	RT (min)	*m*/*z*	Annotation	Adduct Type	Fold Change	Raw *p*	FDR	AUC
1600	7.195	464.3049	Glycohyocholic acid	[M−H]^−^	2.0712	0.0938	Ns	0.6015
2515	7.272	512.2726	Sulfolithocholylglycine	[M−H]^−^	0.4032	0.0192	Ns	0.5846
5195	9.226	658.485	ΔPE(o13:0/18:3)	[M+H]^+^	0.5019	0.0018	q < 0.05	0.7692

Footnote: Estimated compound names are indicated for Rank A annotations. Compound names prefixed with Δ denote Rank B annotations. Fold change represents the rejection/non-rejection ratio. *p*-values (univariate Welch’s *t*-test) are uncorrected for multiple testing. Metabolites showing significant differences (q < 0.05) after applying the Benjamini–Hochberg false discovery rate (FDR) correction are marked. AUC is the area under the ROC curve. PE, phosphatidylethanolamine; (18:3), fatty acid with 18 carbons and 3 double bonds; o13:0, ether-linked fatty acid (13:0); RT, retention time; Ns, not significant; Δ, Rank B annotation.

**Table 4:** Characteristics of the eight serum metabolites selected for the final combined LASSO regression model with one biochemical parameter for RAI prediction.

Peak ID	RT (min)	*m*/*z*	Annotation	Adduct Type	Fold Change	Raw *p*	FDR	Spearman ρ
955	8.427	311.2252	FA(18:2)+2O	[M−H]^−^	1.8951	0.0042	q < 0.05	0.4616
5097	13.241	746.5182	ΔPE(p38:6)	[M−H]^−^	1.4902	0.0288	q < 0.05	0.3817
5304	14.095	774.5488	ΔPE(p40:6)	[M−H]^−^	1.5161	0.0276	q < 0.05	0.4226
2284	0.851	279.1974	ΔCarnitine related	[M+H]^+^	1.9721	0.0529	ns	0.4428
467	1.576	150.061	Methionine	[M+H]^+^	1.5545	0.0186	q < 0.05	0.4365
2069	0.937	263.1517	Creatine	[2M+H]^+^	2.3512	0.0090	q < 0.05	0.3839
725	0.813	170.0455	Unannotated	[M+H]^+^	2.2620	0.0259	q < 0.05	0.2569
1273	3.048	203.976	Unannotated	[M+H−H_2_O]^+^	1.5516	0.0012	q < 0.05	0.5570

Footnote: Estimated compound names are indicated for Rank A annotations. Compound names prefixed with Δ denote Rank B annotations. Fold change represents the rejection/non-rejection ratio. *p*-values (univariate Welch’s *t*-test) are uncorrected for multiple testing. Metabolites showing significant differences (q < 0.05) after applying the Benjamini–Hochberg false discovery rate (FDR) correction are marked. Spearman correlation coefficient (r) for RAI. PE, phosphatidylethanolamine; FA, fatty acid; (18:2), fatty acid with 18 carbons and 2 double bonds; FA(18:2)+2O, oxidized form of FA(18:2) with two oxygen atoms added; p38:6, plasmalogen-type phospholipid with 38 carbons and 6 double bonds; RT, retention time;; ns, not significant; Δ, Rank B annotation.

## Data Availability

The original contributions presented in this study are included in the article/Supplementary Material. Further inquiries can be directed to the corresponding author.

## Supplementary Materials

The following supporting information can be downloaded at: https://www.mdpi.com/article/10.3390/metabo16040273/s1, Supplemental digital content (SDC), Supplementary Methods; Figure S1: Leave-One-Out, Permutation and “0.632+ Bootstrap” Validation of the Three-Variable Logistic Regression Model (POD1-Bile); Figure S2: Compound Annotations for Bile Metabolites in the Three-Variable Logistic Regression Model; Figure S3: Leave-One-Out, Permutation and “0.632+ Bootstrap” Validation of the Nine-Variable LASSO Regression Model (POD14-Serum) with GGT Change; Figure S4: Compound Annotations for Serum Metabolites in the LASSO Regression Models; Figure S5: ROC Curves and Box Plots for Bile Metabolites in the Three-Variable Logistic Regression Model; Figure S6: Scatter Plots for Serum Metabolites in the LASSO Regression Models. Table S1: Twenty-two Bile Metabolites Selected Using PLS-DA; Table S2: Thirty-Eight Serum Me-tabolites Selected Using PLS-DA.

## Abbreviations

AR	acute rejection
DEAS	dehydroepiandrosterone sulfate
GGT	γ-glutamyl transferase
LASSO	least absolute shrinkage and selection operator
LC-QTOF-MS	liquid chromatography quadrupole time-of-flight mass spectrometry
LOOCV	leave-one-out cross validation
LPC	lysophosphatidylcholine
LT	liver transplantation
MS	mass spectrometry
MSE	mean squared error
PLS-DA	partial least squares discriminant analysis
POD	post-operative day
QC	quality control
RAI	rejection activity index
RSD	relative standard deviation
VIP	variable importance in projection
