# Supplementary material for: Bile and Serum Metabolomics in Living Donor Liver Transplantation: Exploratory Insights into Acute Rejection Biomarkers
Source: Metabolites. 2026 Apr 17;16(4):273. doi: 10.3390/metabo16040273 (PMC13117272; doi:10.3390/metabo16040273)
Supplement: Supplementary file 1 [file metabolites-16-00273-s001.zip › metabolites-4243067-supplementary.pdf]

Bile and Serum Metabolomics in Living Donor Liver Transplantation: Exploratory Insights into Acute Rejection Biomarkers

Supplementary Materials

Supplementary method
Supplementary Figures S1- S6
Supplementary Tables S1-S2

Supplementary method

Chemicals and Reagents

Methanol (MeOH), acetonitrile, 2-propanol, ultra-pure water (UPW), and formic acid were purchased from FUJIFILM Wako Pure Chemical (Osaka, Japan) and were of LC-MS grade. L-phenylalanine-13C6 was obtained from MedChemExpress (Monmouth Junction, NJ, USA, #HY-125731S). Sulfadimethoxine was purchased from FUJIFILM Wako Pure Chemical (#190-11101). Fusidic acid was obtained from Tokyo Chemical Industry (Tokyo, Japan, #F1007), 16:0-d31-18:1 phosphatidyletanolamine (PE(34:1)d31) was procured from Avanti Research (Alabaster, AL, USA, #860374P).

Fig. S1

(A)

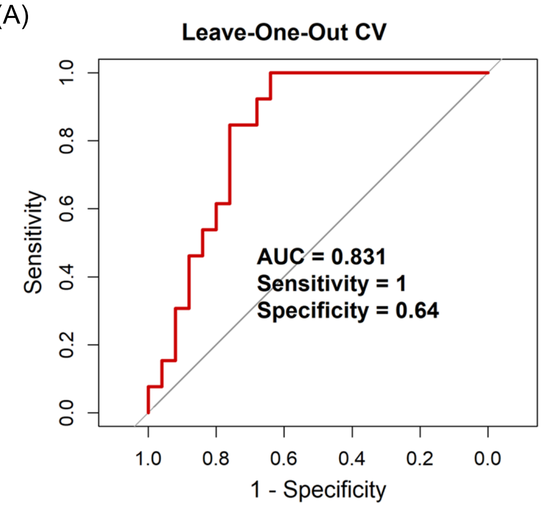

Supplementary Figure S1. Leave-One-Out, Permutation and ".632+ Bootstrap" Validation of the Three-Variable Logistic Regression Model (POD1-Bile)

(A) Leave-One-Out cross-validation with 1,000 iteration yielding AUC of 0.831. The ROC curve is indicated by red line. (B) Permutation test with 1,000 iterations showing the null distribution of AUC values under random class labels. The observed AUC (0.886) is indicated by the vertical red line, yielding an empirical p value of 0.002.(C) ".632+ Bootstrap" with 1,000 iterations showing the distribution of AUC values. The 95% confidence interval for the AUC was 0.806–0.898, indicating moderate internal stability of the model. Model building and validation were performed using R (ver. 4.4.2).

(B)

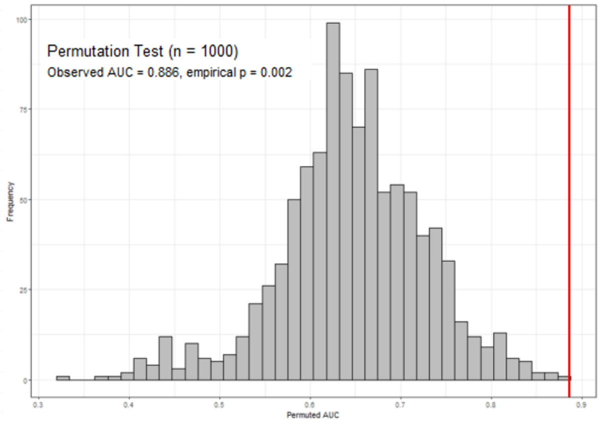

(C)

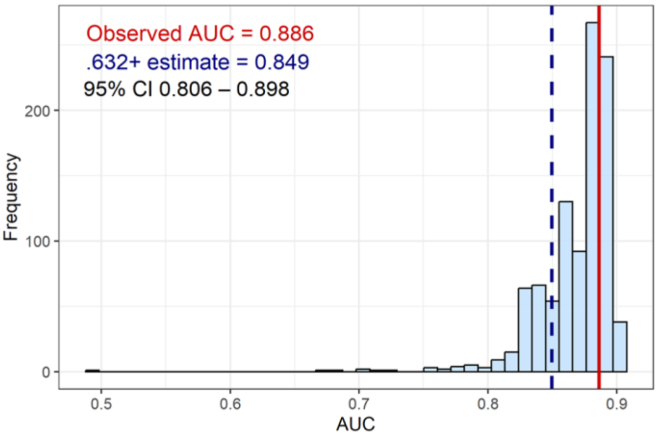

Fig. S2

X1600
[M-H]-

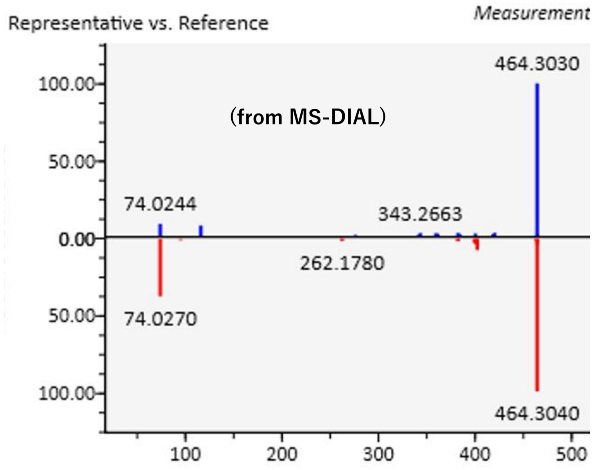

Glycohyocholic acid

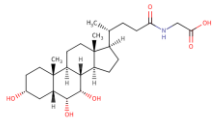

X2515
[M-H]-

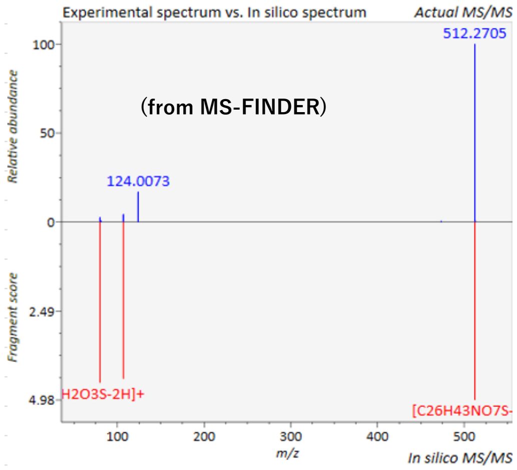

Sulfolithocholylglycine

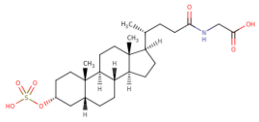

X5195
[M+H]+

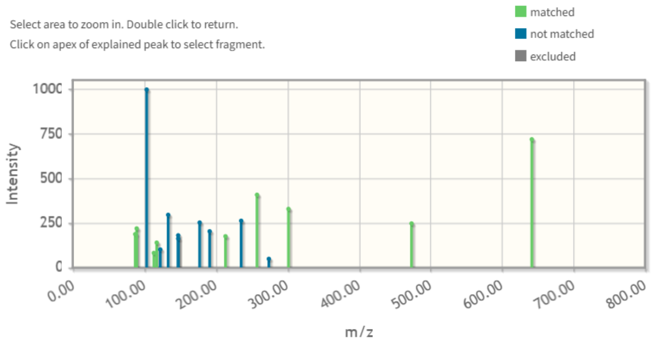

(from MetFrag)

ΔPE(o13:0/18:3)

Supplementary Figure S2. Compound Annotations for Bile Metabolites in the Three-Variable Logistic Regression Model

Compound annotations were performed based on MS/MS spectra using MS-DIAL (ver.5.1), MS-FINDER (ver.3.56), and MetFrag (http://msbi.ipb-halle.de/MetFragBeta/). Metabolite X1600 was annotated as glycohyocholic acid, the minor isomer among three presumed glycocholic acid isomers, eluting earliest. Metabolite X5195 was annotated as ether-type PE(o13:0/18:3), with related compounds such as plasmalogen-type PE(p13:0/18:2) also possible. Δ, provisionally annotated as Rank B.

Fig. S3

(A)

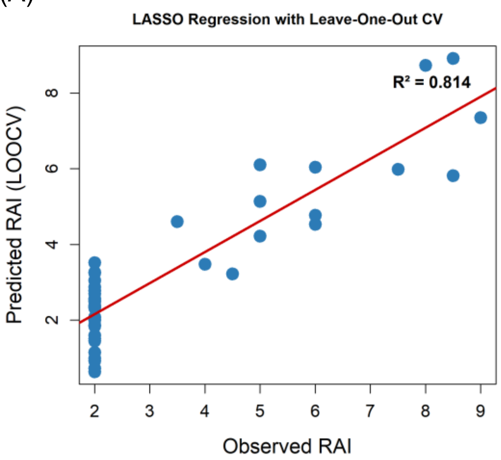

Supplementary Figure S3. Leave-One-Out, Permutation and “.632+ Bootstrap” Validation of the Nine-Variable LASSO Regression Model (POD14-Serum) with GGT Change

(A) Leave-One-Out cross-validation with 1,000 iteration yielding R^2 of 0.814. The regression line is indicated by red line. (B) Distribution of R^2 values obtained from permutation testing (n = 200). The observed R^2 is 0.752, yielding an empirical p value of 0.000. (C) Distribution of R^2 values obtained from “.632+ Bootstrap” (n = 1,000). The red vertical line indicates the observed R^2 of the original model. The 95% confidence interval for R^2 ranged from 0.739 to 0.896, indicating relatively high predictive performance with limited stability. Model building and validation were performed using R (ver. 4.4.2).

(B)

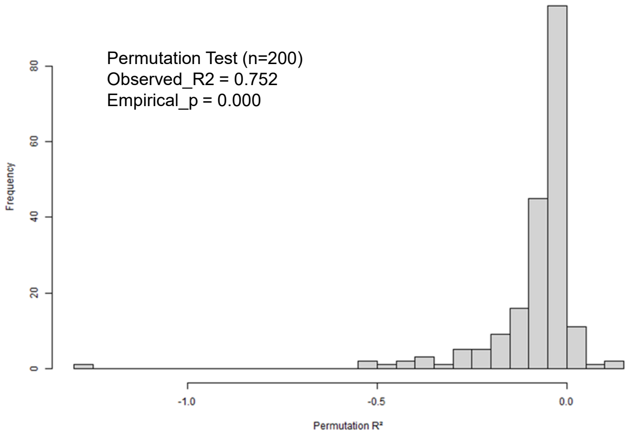

(C)

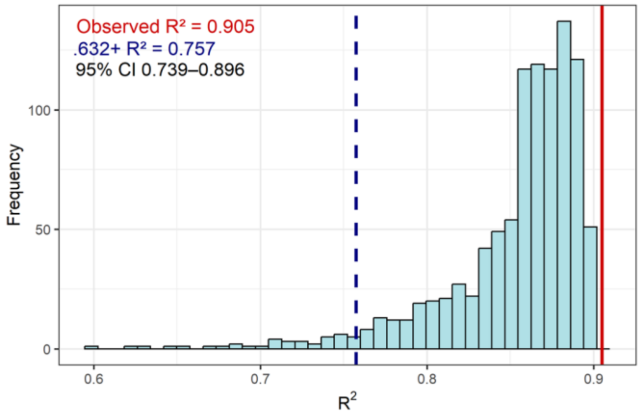

Fig. S4

X467
[M+H]+

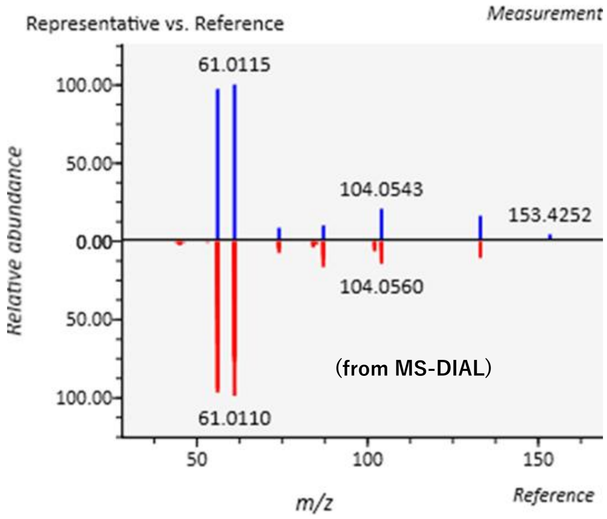

Methionine

X955
[M-H]-

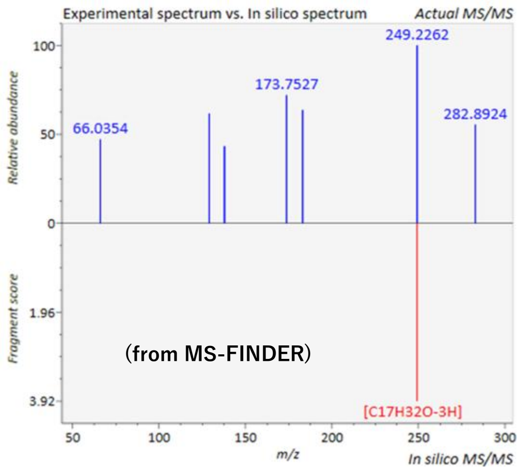

9,10DiHODE
FA(18:2)+2O

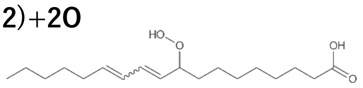

X2069
[2M+H]+

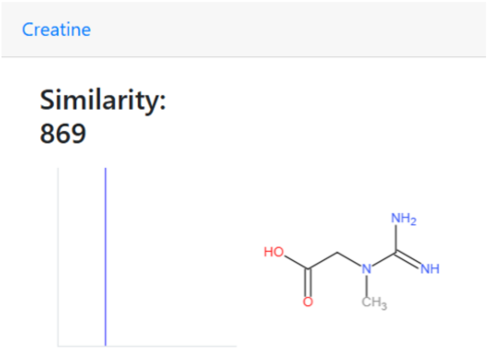

(from MassBank of North America)

Creatine

X5097
[M+H]+

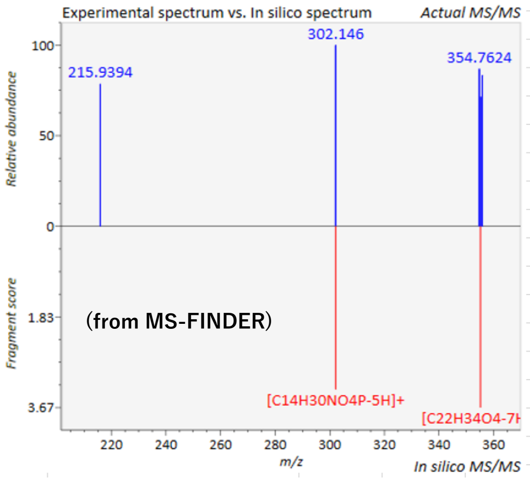

ΔPE(p38:6)

X4759
[M+H]+

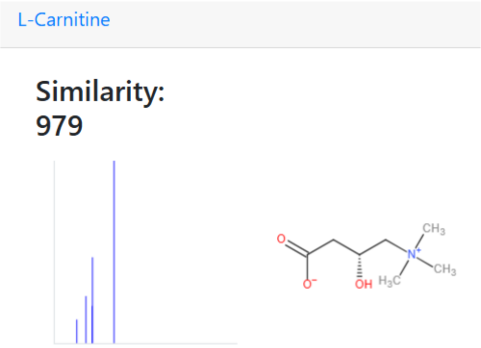

(from MssBank of North America)

Δ Carnitine related

X5304
[M-H]-

No MS2 data

Formula	Error [mDa	Error [ppm	Score	Resource
Spectral DB searc	0.0000	0.0000	5.0000	
C45H78NO7P	-4.5295	-5.8404	1.7910	HMDB,ChEBI,Fool

(from MS-FINDER)

Δ PE(p40:6)

Supplementary Figure S4. Compound Annotations for Serum Metabolites in the LASSO Regression Models

Compound annotations were performed based on MS/MS spectra using MS-DIAL (ver.5.1), MS-FINDER (ver.3.56), and MassBank of North America (https://mona.fiehnlab.ucdavis.edu/spectra/search). Metabolite X4759 was annotated as a carnitine-related compound based on high-confidence MS/MS spectral matching to carnitine in MassBank of North America. Δ, provisionally annotated as Rank B.

Fig. S5

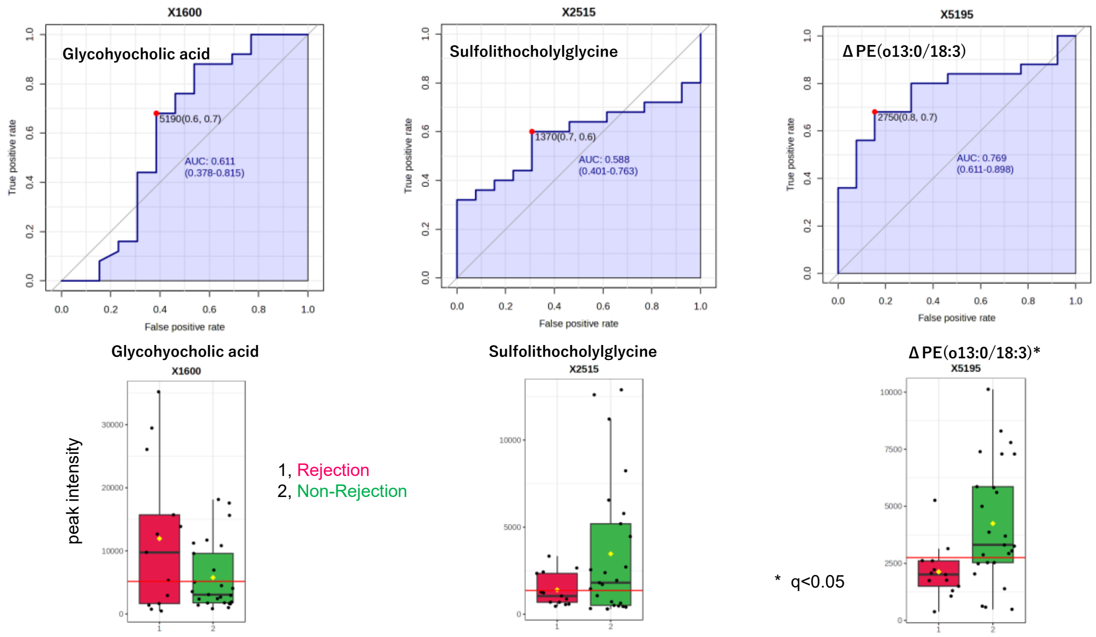

Supplementary Figure S5. ROC Curves and Box Plots for Bile Metabolites in the Three-Variable Logistic Regression Model

ROC curves and box plots for the three metabolites in the logistic regression model, derived from POD1-bile metabolomics data, are shown. Figures were generated using MetaboAnalyst (ver.6.0, https://www.metaboanalyst.ca/MetaboAnalyst/, accessed August 23, 2025).

Fig. S6

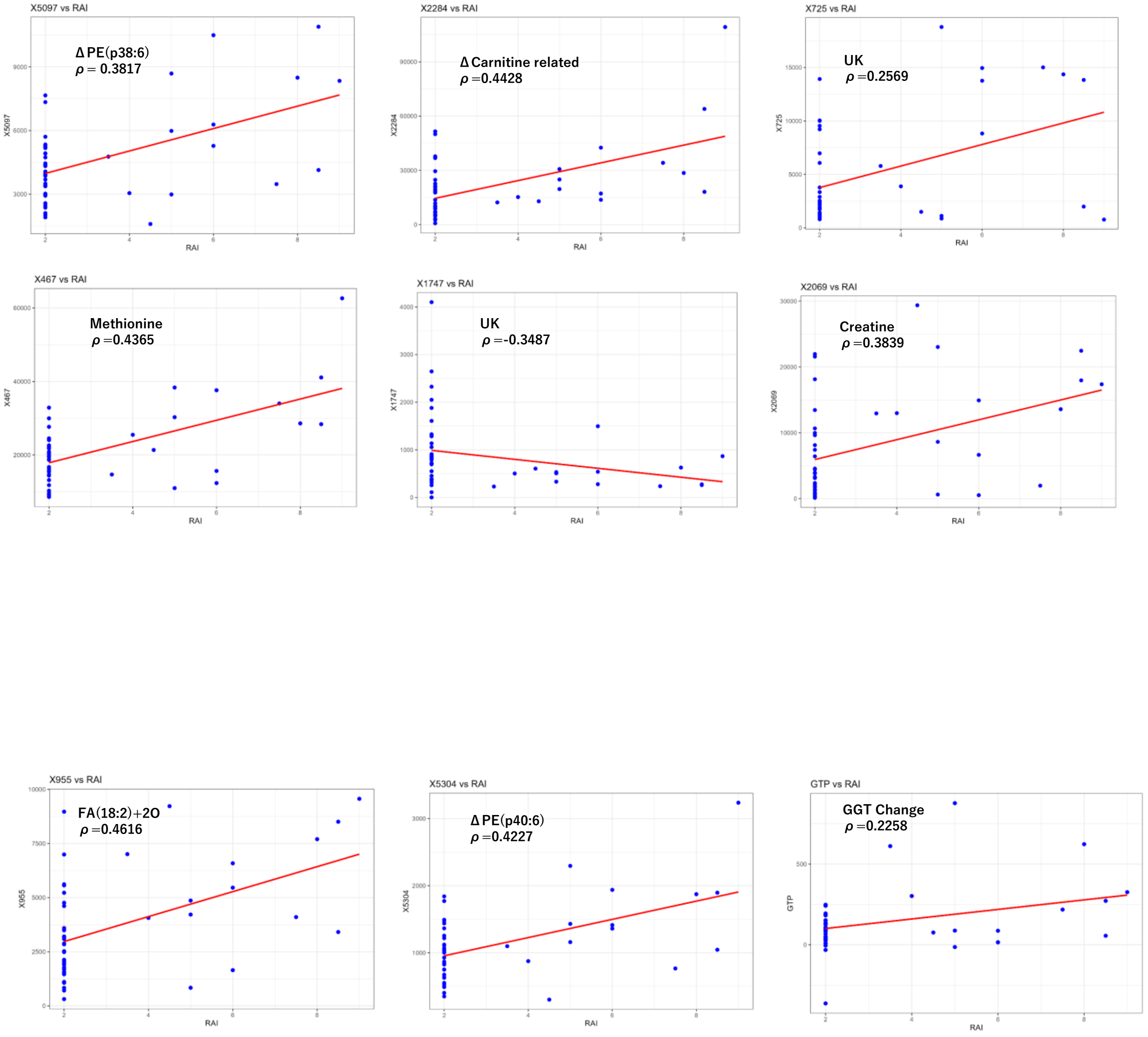

Supplementary Figure S6. Scatter Plots for Serum Metabolites in the LASSO Regression Models

Scatter plots for the 11 metabolites in the LASSO regression model, derived from POD14-serum metabolomics data, are shown with regression lines depicted as red lines. Spearman correlation coefficient values ρ are shown. A correlation of the changes in GGT change (POD14-POD1) with RAI is additionally shown. Figures were generated using R (ver.4.4.2).

Supplementary Table 1. Twenty-two Bile Metabolites Selected Using PLS-DA

ID	RT (min)	m/z	Annotation	Adduct Type	Fold Change	Raw p	FDR	AUC
2628	8.172	514.29	Taurocholic acid	[M-H]-	0.2333	0.0142	ns	0.6031
1665	7.674	466.3113	Unannotated	[M+FA-H]-	0.6464	0.0197	ns	0.6677
4437	14.073	804.5819	PC(16:0/18:1)	[M+FA-H]-	0.5735	0.0962	ns	0.6462
2974	8.029	530.2836	Unannotated	[M+FA-H]-	0.1865	0.0563	ns	0.6246
1600	7.195	464.3049	Glycohyocholic acid	[M-H]-	2.0712	0.0938	ns	0.6015
4522	13.108	826.5666	ΔPC(36:4)	[M+FA-H]-	0.5196	0.0846	ns	0.6523
4323	7.88	773.3092	Unannotated	[M-H]-	2.6400	0.0999	ns	0.6677
3743	8.282	614.2135	ΔHematoporphyrin related	[M+FA-H]-	0.5833	0.0098	ns	0.7046
4341	13.213	776.5502	PC(16:1/16:0)	[M+FA-H]-	0.5464	0.0899	ns	0.6431
4580	12.87	850.5664	ΔPC(38:6)	[M+FA-H]-	0.4404	0.0498	ns	0.6892
3585	8.886	597.2753	Hematoporphyrin	[M-H]-	1.8100	0.0626	ns	0.6985
2515	7.272	512.2726	Sulfolithocholylglycine	[M-H]-	0.4032	0.0192	ns	0.5846
3707	8.517	611.2916	Unannotated	[M-H]-	3.9463	0.0888	ns	0.6769
3587	8.783	597.2758	Hematoporphyrin	[M-H]-	1.7989	0.0674	ns	0.6892
3960	7.112	651.39	ΔN-Acetylglucosaminyl ursodeoxycholyl glycine	[M-H]-	3.6047	0.0579	ns	0.6677
3579	7.675	488.3074	Glycocholic acid	[M+Na]+	0.6132	0.0310	ns	0.6615
5025	8.182	629.309	ΔHematoporphyrin related	[M+H]+	2.9436	0.0491	ns	0.6892
5195	9.226	658.485	ΔPE(o13:0/18:3)	[M+H]+	0.5019	0.0018	q<0.05	0.7692
5359	9.229	702.5125	Unannotated	[M+H]+	0.5404	0.0031	q<0.05	0.7415
5752	12.603	780.5672	PC(36:5)	[M+H]+	0.4896	0.0409	ns	0.6585
4122	12.66	536.1758	Unannotated	[M+H]+	1.4316	0.0786	ns	0.6400
5831	14.236	786.6156	PC(36:2)	[M+H]+	0.5760	0.0808	ns	0.6646

Footnote: Estimated compound names are indicated for Rank A annotations. Compound names prefixed with Δ denote Rank B annotations. Fold change represents the rejection/non-rejection ratio; values >2 are highlighted in red, and values <0.5 are highlighted in blue. p-values (univariate Welch's t-test) are uncorrected for multiple testing; p-values <0.05 are highlighted in green, and p-values <0.01 are highlighted in red. Metabolites showing significant differences (q<0.05) after applying the Benjamini-Hochberg false discovery rate (FDR) correction are marked. AUC is the area under the ROC curve; values are >0.7 highlighted in red. PE, phosphatidylethanolamine; PC, phosphatidylcholine; FA, fatty acid; (18:2), fatty acid with 18 carbons and 2 double bonds; (36:4), fatty acid with 36 carbons and 4 double bonds; o13:0, ether-linked fatty acid (13:0); RT, retention time; ns, not significant. The three metabolites selected in the binary prediction model for rejection using logistic regression are highlighted with orange.

Supplementary Table 2. Thirty-Eight Serum Metabolites Selected Using PLS-DA

Peak ID	RT (min)	m/z	Annotation	Adduct type	Fold Change	Raw p	FDR	Spearman ρ
4948	13.392	714.5121	PE(16:0/18:2)	[M-H]-	1.7613	0.0133	q<0.05	0.3692
4206	11.295	583.2606	Bilirubin	[M-H]-	1.6561	0.0764	ns	0.2442
5035	13.161	738.5131	PE(16:0/20:4)	[M-H]-	1.4179	0.0415	ns	0.2004
5251	14.027	766.545	PE(18:0/20:4)	[M-H]-	1.4326	0.0254	ns	0.3484
5481	12.81	800.551	PC(16:0/18:3)	[M+HCOO]-	1.7774	0.0645	ns	0.3206
198	0.821	146.0466	Glutamic acid	[M-H]-	2.5628	0.0243	ns	0.3111
955	8.427	311.2252	FA(18:2)+2O	[M-H]-	1.8951	0.0042	q<0.05	0.4616
5058	14.327	742.5446	PE(18:0/18:2)	[M-H]-	1.5785	0.0136	q<0.05	0.3936
5097	13.241	746.5182	ΔPE(p38:6)	[M-H]-	1.4902	0.0288	q<0.05	0.3817
4202	11.186	583.2592	Bilirubin minor	[M-H]-	3.1748	0.0704	ns	0.2860
4349	8.893	597.2766	ΔHematoporphyrin	[M-H]-	2.4303	0.0816	ns	0.2947
4177	7.639	581.2449	Biliverdin	[M-H]-	2.0271	0.0815	ns	0.3193
393	0.813	193.0356	Glucuronic acid	[M-H]-	1.8030	0.0905	ns	0.3493
129	0.817	128.036	5-Oxoproline	[M-H]-	2.4911	0.0181	q<0.05	0.4109
894	7.624	301.1217	Unannotated	[M-H]-	2.2182	0.0719	ns	0.3340
937	8.213	309.2089	ΔFA(18:3)+2O	[M-H]-	2.1971	0.0380	ns	0.4395
5304	14.095	774.5488	ΔPE(p40:6)	[M-H]-	1.5161	0.0276	q<0.05	0.4226
1385	7.627	367.1608	Dehydroepiandrosterone sulfate	[M-H]-	0.6490	0.0689	ns	-0.1461
4275	8.871	590.3508	LPC(20:3)	[M+HCOO]-	0.6400	0.0171	q<0.05	-0.2737
1747	8.28	397.2076	Unannotated	[M-H]-	0.5088	0.0077	q<0.05	-0.3487
2284	0.851	279.1974	ΔCarnitine related	[M+H]+	1.9721	0.0529	ns	0.4428
9581	8.034	761.3183	Unannotated	[M+H]+	2.2683	0.0949	ns	0.3157
467	1.576	150.061	Methionine	[M+H]+	1.5545	0.0186	q<0.05	0.4365
7685	7.007	585.2828	Unannotated	[M+H]+	1.5088	0.0932	ns	0.2373
2069	0.937	263.1517	Creatine	[2M+H]+	2.3512	0.0090	q<0.05	0.3839
1290	3.045	204.9851	Unannotated	[M+H]+	1.7511	0.0013	q<0.05	0.5655
7872	8.892	598.2897	Unannotated	[M+H]+	1.9630	0.0842	ns	0.2621
725	0.813	170.0455	Unannotated	[M+H]+	2.2620	0.0259	q<0.05	0.2569
89	0.904	104.0722	Δ2-Aminobutyric acid	[M+H]+	1.9729	0.0033	q<0.05	0.5369
577	3.045	161.0652	Δ6-Methylcoumarin	[M+H]+	2.0404	0.0023	q<0.05	0.5529
1273	3.048	203.976	Unannotated	[M+H-H2O]+	1.5516	0.0012	q<0.05	0.5570
52	0.792	90.0564	Alanine	[M+H]+	1.4548	0.0036	q<0.05	0.5235
80	0.815	102.0567	Unannotated	[M+H-H2O]+	1.6205	0.0158	q<0.05	0.4021
4048	0.83	382.0465	Unannotated	[M+H]+	1.8883	0.0140	q<0.05	-0.2443
2879	6.069	313.1611	ΔPhenylanalylphenylalanine	[M+H]+	0.3731	0.0321	ns	-0.5359
817	6.154	177.0792	Unannotated	[M+H]+	0.5312	0.0397	ns	-0.2669
4759	5.11	418.2019	Fibrinopeptide_A(30-34)	[M+H]+	0.4576	0.0005	q<0.05	-0.5462
3732	7.044	363.224	ΔCortisol	[M+H]+	0.3785	0.0001	q<0.05	-0.5635

Footnote: Estimated compound names are indicated for Rank A annotations. Compound names prefixed with Δ denote Rank B annotations. Fold change represents the rejection/non-rejection ratio; values >2 are highlighted in red, and values <0.5 are highlighted in blue. p-values (univariate Welch's t-test) are uncorrected for multiple testing; p-values <0.05 are highlighted in green, and p-values <0.01 are highlighted in red. Metabolites showing significant differences (q<0.05) after applying the Benjamini-Hochberg false discovery rate (FDR) correction are marked. Spearman correlation coefficient (r) for RAI; values >0.5 are highlighted in red. PE, phosphatidylethanolamine; PC, phosphatidylcholine; FA, fatty acid; (18:2), fatty acid with 18 carbons and 2 double bonds; FA(18:2)+2O, oxidized form of FA(18:2) with two oxygen atoms added; p38:6, plasmalogen-type phospholipid with 38 carbons and 6 double bonds; RT, retention time; AUC, area under the ROC curve; ns, not significant. Eight metabolites selected in the continuous variable prediction model for RAI using LASSO regression are highlighted with blue.
